# Transcriptomic Profiling of the Allorecognition Response to Grafting in the Demosponge *Amphimedon queenslandica*

**DOI:** 10.3390/md15050136

**Published:** 2017-05-11

**Authors:** Laura F. Grice, Bernard M. Degnan

**Affiliations:** School of Biological Sciences, University of Queensland, Brisbane QLD 4072, Australia; laura.grice@uqconnect.edu.au

**Keywords:** allorecognition, histocompatibility, invertebrates, Porifera, RNA-Seq, self-nonself recognition, sponge

## Abstract

Sponges, despite their simple body plan, discriminate between self and nonself with remarkable specificity. Sponge grafting experiments simulate the effects of natural self or nonself contact under laboratory conditions. Here we take a transcriptomic approach to investigate the temporal response to self and nonself grafts in the marine demosponge *Amphimedon queenslandica*. Auto- and allografts were established, observed and sampled over a period of three days, over which time the grafts either rejected or accepted, depending on the identity of the paired individuals, in a replicable and predictable manner. Fourteen transcriptomes were generated that spanned the auto- and allograft responses. Self grafts fuse completely in under three days, and the process appears to be controlled by relatively few genes. In contrast, nonself grafting results in a complete lack of fusion after three days, and appears to involve a broad downregulation of normal biological processes, rather than the mounting of an intense defensive response.

## 1. Introduction

Marine benthic ecosystems often are densely-populated and have remarkable levels of animal biodiversity (e.g., coral reefs). In such a crowded environment, space can become a limiting resource, and sessile invertebrates in particular often face intense competition for habitat and growth space. For example, one study determined that 42% of microhabitats (i.e., gastropod shells) for the colonial hydrozoan *Hydractinia echinata* must be shared between two or more colonies [1]. Similar population crowding has been observed at Woods Hole, Massachusetts, where multi-individual clumps of the sponge *Clathria prolifera* were identified at relatively high (20%) frequencies within the population [2]. Such crowding means the chance of direct contact between individuals of the same or different species—conspecifics or xenospecifics, respectively—is high. Fusion may at times be beneficial, for example by allowing an individual to re-fuse with itself following fragmentation or growth around an object, or through increased survivorship and subsequent reproductive output associated with increased size [3,4,5]. However, there is often a cost associated with conspecific fusion, since individuals within a chimera are at risk of parasitism whereby the stem cells of one fusion partner gain disproportionate access to the germ line and monopolise reproductive output [6]. For this reason, fusion is generally limited to genetically-identical individuals or close kin [7]. The decision to fuse with or reject a potential partner is mediated by the allorecognition (i.e., self-nonself recognition) system.

The sponge has been a useful model animal for the study of cell adhesion and self-nonself recognition for almost 150 years, with grafting experiments first described in 1869 [8]. Sponge grafts aim to experimentally emulate the effects of natural self or nonself sponge-sponge contact. Grafting is performed by apposing two pieces of sponge, either from different parts of the same sponge (autograft) or from two different sponges of the same (allograft) or different (xenograft) species. These experiments have demonstrated that sponges are capable of distinguishing between self and nonself see for example [2,9,10,11,12,13,14,15,16,17,18,19]. Fusion is limited almost exclusively to autografts, although fusion between different sponge individuals has been observed in rare cases at rates inversely proportional to the physical distances between sponge graft partner habitats [11,13,18]. This trend can be explained broadly by the general decrease in genetic similarity between individuals with increasing distance [11,20].

Typical self grafts that undergo fusion are characterised by the breakdown of the pinacoderm layers separating the two pieces of sponge, with the interface between the graft donors becoming invisible over time [2,15,21]. Responses to allografts, however, vary extensively even within a single sponge genera [22]. Reactions can be fast, such as in *Clathria prolifera*, which responds to allografting in two to six hours [2,23], or comparatively slow, as in *Callyspongia diffusa*, which can take up to a week to react [2,10,20,24,25,26]. Processes that characterise graft rejection may include cellular necrosis of one or both graft partners [2,10,20,24,25], collagen deposition to form a physicochemical barrier between the apposing sponges [2,12,22,23,27,28,29], cellular migration to the point of contact [20,21,22,23,29,30], and phagocytic or cytotoxic reactions [22,24,25,26]. Qualitative and quantitative responses to grafts are replicable and predictable [2,24,31], between both first-party (sponge A:B replicates) and third-party (where A:B fusion predicts identical A:C and B:C reactions) grafts [13,25,28]. This specificity and repeatability indicates that recognition responses are governed by an underlying polymorphic genetic system, rather than by environmental or random effects [20,32].

Here we analyse the expression dynamics of the auto- and allograft response in the demosponge *Amphimedon queenslandica*. We established and observed grafts over a three-day period, over which time each graft underwent either a fusion or a rejection reaction. We periodically took cellular samples from the graft interface. Transcriptomes generated from these samples were analysed to identify the broader functional changes that distinguish the self grafting response from the nonself grafting response in *A. queenslandica*. To our knowledge, this represents the first longitudinal, high-throughput sequencing approach applied to understanding the molecular allorecognition response in sponges.

## 2. Results

Sponge grafting experiments have been well-described in the literature since 1869 [8]. However, advances in DNA and RNA sequencing technologies mean that the sponge graft response can now be studied on a transcriptome-wide scale. We performed a classical self and nonself grafting experiment between *A. queenslandica* individuals, and analysed the qualitative and quantitative changes in expression that occurred across the graft time course.

### 2.1. Physiological Responses to Grafting

Grafts were established between four pairs of sponge individuals, with each pairing producing one nonself and two self (i.e., one self time course per sponge) time courses. Multiple grafts were created to allow separate analysis at each time point to avoid disturbance during observation. The grafts were observed at 12, 24, 48 and 72 h post grafting (hpg) to determine the nature and timing of the physiological response to self or nonself contact in *A. queenslandica*. Samples were collected from the graft interface at each time point.

#### 2.1.1. Autografts

**Figure 1:**
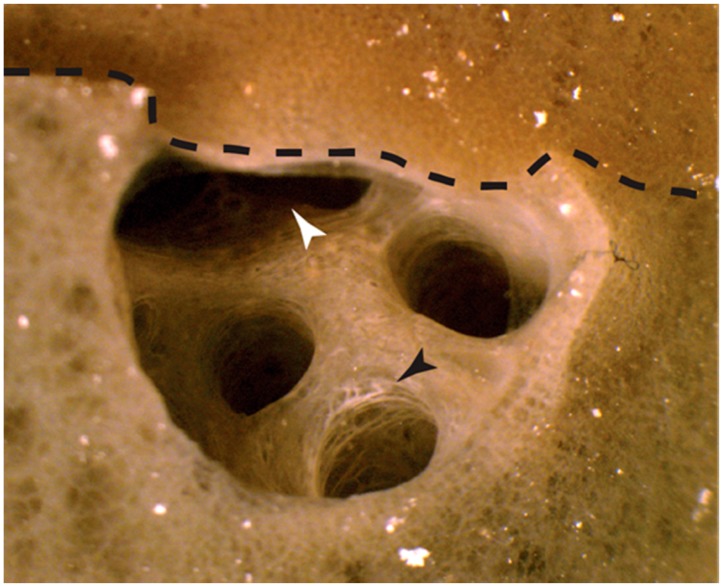
Remodelling of an osculum following self-grafting. An internal osculum bisected during graft preparation and placed at the autograft interface triggered the adjacent self-sponge piece to remodel to form a continuous chamber inside the fused sponge by 72 h post grafting (hpg). Black arrow—white cellular region can be seen at the cut surface of the osculum that was not in contact with self and signs of sponge healing are apparent by 72 hpg. White arrow—indicates where the chamber continued into the other half of the sponge autograft. Visual inspection of the chamber revealed that it continued deep inside the sponge.

For six of the eight autograft time courses, early signs of fusion were first observed at 12 hpg. Grafts from the seventh sponge initiated fusion by 24 hpg, and the eighth by 48 hpg (Table 1). Bonds between sponge pieces grew progressively stronger as the fusion progressed, with all self samples unambiguously fused by 48 hpg. Typically, by 72 hpg the two sponge pieces could not be separated with reasonable force, and the line dividing the pieces was difficult to observe without microscopy. Signs of cellular remodelling were also observed by 72 hpg. For example, in one sample, a bisected osculum originally sat on one side of the point of fusion, and by 72 hpg both sides of the graft appeared to have remodelled to develop a new chamber (Figure 1).

#### 2.1.2. Allografts

Twelve hours after grafting, all four allograft samples remained unfused. However, at 24 hpg several of the samples exhibited signs of partial attachment (Table 1). Here, weak fibrous connections were present between apposed sponge slices, although these bonds were easily broken with a light amount of force. By 72 hpg, no fusion between grafted slices was ever observed. Both sponge partners within the grafts appeared alive and healthy, although the cut surfaces at times appeared fibrous and whitened.

### 2.2. Transcriptome Sequencing and Statistics

One of the four graft experiments was selected for whole-transcriptome RNA sequencing (RNA-Seq) and analysis. It comprised of two self and one nonself time courses, each sampled at 0, 12, 24, 48 and 72 hpg. Samples are named here by time point (Donor, T12–T72) and graft type (AA or BB for self, AB for nonself). Final sequencing datasets each contained between 17.5 (T24AB_C) and 27.8 (Donor A) million reads (Table 2). The average GC count per library was 42.3%, which is slightly higher than the genomic average across all *A. queenslandica* genes (38.1% as calculated using the *A. queenslandica* genome data available through BioMart) [33]. Read trimming resulted in the loss of approximately 6% of reads per sample, and shortening of the remaining reads (Table 2).

### 2.3. Principal Component Analysis

**Figure 2:**
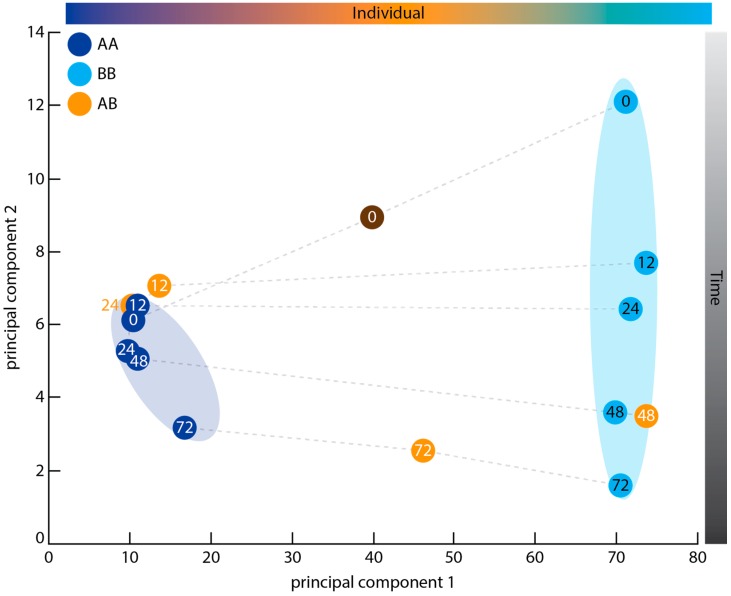
Principal component analysis of dynamically expressed genes. Each circle represents a transcriptome within the graft time courses. Dots are coloured by donor sponge (A/AA—dark blue, B/BB—light blue, AB—orange; Donor AB is coloured grey to indicate that it is an artificial sample formed by merging the Donor A and Donor B sequencing reads) and numbered by time point (0–donor sample, 12–12 h post grafting (hpg), 24–24 hpg, 48–48 hpg, 72–72 hpg). Shaded rings group the A/AA and B/BB samples, respectively. Dashed lines link samples representing the same time point from different time courses. Shaded bars at the top and right sides of the graph summarise the results of the analysis, showing the biological variables that best explain the sample separations observed across each axis. Sample separation is based on the top 0.9th quantile of dynamically expressed genes, as determined by BLIND (“Basic Linear INdex Determination of transcriptomes”; see Materials and Methods).

Genetic identity, rather than immune state, appears to be the primary factor promoting gene expression differences between samples, when considering the most dynamically-expressed genes across all samples. In a principal component analysis (PCA) (Figure 2), the AA and BB autograft samples formed two separate clusters along the first principal component. The autograft samples also showed a chronological separation of samples by hours post grafting along the second principal component. Although both the AA and BB time courses displayed this trend, the AA samples formed a tight cluster while the BB samples spread out across the second principal component axis (Figure 2). The AB allogeneic samples did not cluster along either principal component; instead, individual AB samples tended to group with similarly-staged samples from either the AA or BB time courses (Figure 2). T12AB and T24AB sat with the AA cluster, while T48AB fell close to T48BB. T72AB fell mid-way between the two clusters on the first principal component, and aligned with T72AA and T72BB along the second principal component. It is notable that this middle position of T72AB is also occupied by the artificial Donor AB sample, which was formed by merging the sequencing reads from Donor A and B and included to provide a baseline for expression prior to allografting. In summary, therefore, the first principal component appears to separate the samples by sponge individual, with samples from matching time points sitting in the same general region of the second principal component axis as one another. The arrangement of time points along this second axis is in approximate chronological order (Figure 2).

### 2.4. Differential Gene Expression

RNA-Seq reads from all graft samples were mapped back to the *A. queenslandica* genome to determine the read counts per Aqu2.1 gene model. These counts were then used to identify genes exhibiting statistically significant fold changes between successive pairs of time points. The two self time courses, AA and BB, were analysed separately in light of the finding that between-individual differences were the primary source of variance between samples (Figure 2). For this reason, and the small sample size available, we chose a strict fold change selection threshold: four-fold or greater (log2) changes in expression between successive pairs of time points. In total, we identified 2502 *A. queenslandica* genes with dynamic expression across the graft timecourse.

**Figure 3:**
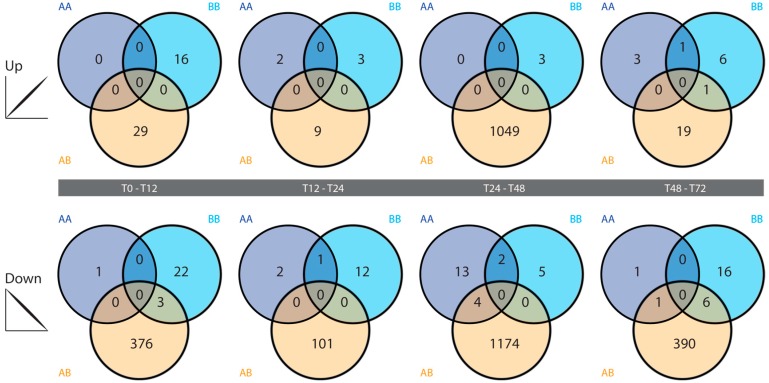
Differentially expressed gene counts. Each Venn diagram shows the number of differentially expressed genes that are up- (top) or down-regulated (bottom) with an observed fold change of 4-fold or greater between pairs of successive time points, in the AA (dark blue), BB (light blue) and/or AB (orange) time courses.

**Figure 4:**
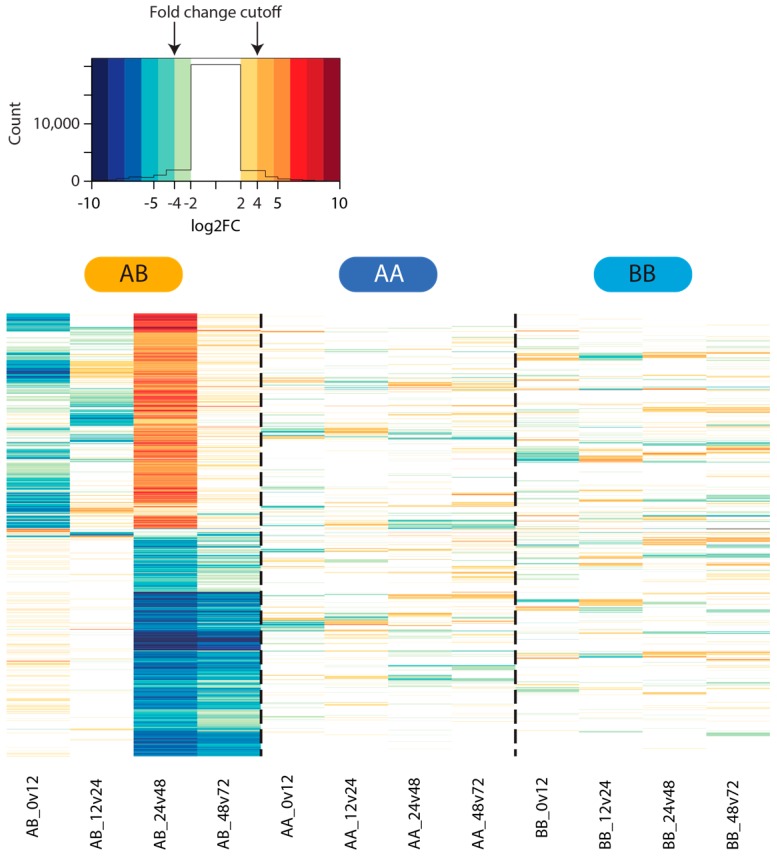
Differential gene expression in the nonself graft time course. Heat map showing the log2 fold changes in expression across the self and nonself graft time courses. Only genes found to be statistically differentially expressed, and exhibiting a 4+-fold expression change, in one or more pairs of nonself time points are shown.

All tested comparisons in the two self time courses exhibited low numbers of statistically significant differentially expressed genes at the filtering threshold used, with little overlap between gene lists from the AA and BB time courses. Greater numbers of differentially expressed genes were identified in the four nonself comparisons (Figure 3). The highest number of differentially expressed genes in the nonself grafts was identified in the 24–48 hpg category, where over 1000 genes each were up- and down-regulated at 48 hpg relative to 24 hpg (Figure 4). Those genes that were upregulated at 48 hpg also showed a general trend of being downregulated at 12 hpg and 24 hpg relative to the preceding time points, while those that were downregulated at 48 hpg exhibited limited changes in the earlier graft stages (Figure 4). These genes display little activity in the self-graft time courses (Figure 4), as expected based on the differentially expressed gene counts presented in Figure 3.

### 2.5. Gene Ontology Analysis

To explore the sponges’ putative functional response to grafting, each list of differentially expressed genes (Table S1) within the nonself time course was analysed to identify gene ontology (GO) terms, which were statistically significantly enriched amongst the genes of interest relative to the genome as a whole. Treemaps showing these results are presented in Figure S1. In particular, these results reveal that chronological progression of the sponge graft response is associated with the downregulation of genes involved in some key biological processes, including cell signalling, transcription and translation, and molecular transport.

## 3. Discussion

### 3.1. Physiological Self and Nonself Graft Responses in Amphimedon queenslandica

The autograft fusion-allograft rejection phenomenon has been well-characterised in sponges (see Introduction), and our morphological results are consistent with past findings. Of the eight examined self graft time courses, fusion was observed for all samples by 48 hpg. Observed variability in the onset time of initial fusion likely represents inter-individual variation, but possibly also variability in graft contact surfaces and/or failure to observe early weak bonds between grafted pieces. In all four nonself graft time courses, rejection had occurred by 72 hpg. Sponge pieces in the rejected grafts remained alive and healthy, with no signs of necrosis obvious to the naked eye. Three of the four nonself graft time courses exhibited signs of minor attachment between 12 and 24 hpg. Here, weak bonds appeared to join the two pieces of sponge, and light force was required to separate the two pieces. The bonds between the pieces may not represent true early fusion, but rather, for example, fibrous material produced during graft rejection that randomly interlaced due to proximity of the two sponge pieces. However, it may be that a degree of fusion is required early in the rejection process, to allow cellular infiltration of the graft interface, direct cell-cell contact between cells of the opposing individuals, and subsequent rejection. Such a phenomenon has been reported elsewhere, with tissue-like bridges spanning the nonself graft interface in other sponge species [16,21,24,25,34,35]. Blocking the graft interface with an artificial membrane, permeable to diffusible factors but not cells, has also been shown to inhibit the rejection response in the demosponge *Callyspongia diffusa* [25], further suggesting that direct cell-cell interactions are critical for sponge allorecognition in some species (though reports from other species suggest this might not be a universal requirement [36]). Early fusion has also been observed in the allorecognition response of the colonial hydroid *Hydractinia symbiolongicarpus*, where transitory fusion represents a temporarily fused state in genetically related but ultimately incompatible pairs [37]. A similar process may be occurring in *A. queenslandica*.

### 3.2. Graft Transcriptome Samples Exhibit Greater Between-Individual Than Between-Time Point Variance

PCA of the most dynamically expressed genes across the three graft time courses revealed greater divergence between sponge individuals than between graft conditions (i.e., autograft or allograft) (Figure 2). This between-individual variance was not revealed until after sequencing was complete, and meant that the two self time courses had to be analysed independently rather than treated as biological replicates. To account for this inter-individual variation, we trialled a reduced experimental model in which samples within a time course were treated as replicates of one another in order to calculate a global common dispersion value. This common dispersion value encompasses the self and nonself graft-induced biological variation and was applied to the full design model, in which each time point was considered separately. The use of this value, and the conservative fold change cutoff used for differential expression analysis, means that a small number of differentially expressed genes were identified within the two self graft comparisons.

### 3.3. Differential Gene Expression Analysis

Relatively low numbers of genes were found to be differentially expressed between successive autograft time points. The allograft time course exhibited more dynamic expression across time, particularly between the 24 and 48 hpg time points (Figure 3). It is notable that around these times in the allograft time course, a physical transition occurs in the grafts from a possible transitory fusion state (variably observed between 12 and 48 hpg) to a complete rejection state. It is therefore possible that the large changes in nonself gene expression at this time are functionally related to this physical transition.

Sixty-five percent of differentially expressed genes identified within the nonself time course were found to be downregulated at the point of detection. These downregulated genes were statistically enriched for GO terms associated with key biological processes such as cell signalling, transcription and translation, protein and molecular transport, and other metabolic processes (Figure S1). This may indicate that a key response to nonself grafting is the shutdown of some regular biological processes, rather than a shift to defensive gene expression. Normal cell signalling appears to be downregulated in response to nonself grafting; for instance, genes with functions associated with signalling pathways such as ubiquitin transferase, or GTP or metal ion binding activity were downregulated at 12 hpg relative to the control state (Figure S1), while genes with more generalised cell signalling roles were downregulated at both 48 and 72 hpg relative to the previous time points (Figure S1). However, a suite of other cell signalling genes were also upregulated at 48 hpg, perhaps indicating a shift to rejection signalling processes, or that previously-downregulated cell signalling genes were being reactivated at this time. Curiously, we did not observe statistically significant enrichments for genes associated with cell adhesion, apoptosis or aggregation factor-mediated cell adhesion as has been reported previously see for example [36,38,39,40]. The reasons for this disparity remain unclear. One possibility is that the rapidly evolving nature of allorecognition systems [32,41] has resulted in distinct molecular rejection processes. Additionally, as discussed in the Introduction, the physical grafting response appears to differ between species, which may explain the involvement of different suites of molecules.

A transcriptional shutdown in response to graft rejection has also been reported in microarray analyses of gene expression in the ascidian *Botryllus schlosseri*. In this species, rejection reactions are asymmetric, where one graft partner develops morphological signs of rejection (the ‘rejected’ individual), while the other partner does not (the ‘rejecting’ individual) [42]. Rejected individuals within a *B. schlosseri* graft showed limited gene upregulation relative to the naive state, but extensive downregulation of genes involved in protein biosynthesis, cell structure and motility, and immune function; rejecting individuals showed limited changes relative to the naive state [42]. Here it was hypothesised that the rejected individual undergoes a period of tissue self-destruction, in order to facilitate physical tissue separation from the rejecting individual, and to inhibit interference of this separation process by the immune or tissue healing systems [42]. It is possible that a similar avoidance strategy is in place in *A. queenslandica*. Additionally, although no obvious physiological signs of a ‘rejected/rejecting’ hierarchy have been noted within *A. queenslandica*, it is possible that some molecular, genetic or physiological hierarchy may indeed exist.

## 4. Materials and Methods

### 4.1. Grafting of Adult Sponges

Four grafting experiments were performed in total. Four pairs of non-adjacent *A. queenslandica* adults (‘donors’) from Shark Bay, Heron Island Reef (Great Barrier Reef, Australia) [43] were removed from their rocky substrates and each cut into twelve pieces of similar size (approximately 3 × 1.5 cm). Grafts were prepared by apposing the internal cut surfaces of two pieces of sponge from the same (autograft) or different paired (allograft) individuals, and were held together with sterile syringe needles. To minimise sample handling, separate grafts were established for each time point (i.e., four self grafts from each of the two sponges, and four nonself grafts). The grafts were kept submerged in a tank with flow-through sea water, and exposed to ambient shaded light until they were required. Auto- and allografts were observed at 12, 24, 48 and 72 hpg. At each time point, one graft from each of the nonself and self time courses was retrieved and examined to assess sponge health and fusion state.

### 4.2. RNA Extraction from Grafts

For one graft experiment, samples were taken from the graft interface and preserved in RNA Later (Ambion) for whole-transcriptome sequencing, both from the two donor sponges prior to graft establishment and from the auto- and allografts at each time point. A total of 200 mg sponge was used per RNA extraction (100 mg sponge from each side of the graft interface, where applicable). RNA was extracted using Tri Reagent (Sigma-Aldrich, St. Louis, MO, USA) following manufacturer’s guidelines. RNA quantity and quality was checked using a Qubit 2.0 (Invitrogen by Life Sciences, Carlsbad, CA, USA) and Bioanalyser 2100 (Agilent Technologies, Santa Clara, CA, USA).

### 4.3. Transcriptome Sequencing

RNA was sequenced by Macrogen Inc. (Seoul, Korea) using a polyA-selection, 100 base pair (bp), paired-end, unstranded Illumina HiSeq 2000 protocol. Eighteen libraries were multiplexed into a single Illumina flow cell lane. For the T24AB time point, three different RNA samples were sequenced from the original sponge sample, due to initial concerns about RNA quality and quantity. However, all resulting T24AB samples were deemed to be of sufficient quality for this analysis and their reads were pooled for subsequent analyses. Library quality was assessed using FastQC 0.10.0 (non-interactive mode, run with Java 1.6.0_22) [44], and poor-quality bases and reads were trimmed with Trimmomatic 0.22 [45] using a headcrop length of 13 bp, a sliding window size of 4 bp and average quality of 15, and a minimum read length of 36 bp. Sequencing statistics are provided in Table 2.

### 4.4. Read Mapping and Counting

Trimmed reads were mapped to the *A. queenslandica* Aqu2.1 gene models [46] using the CLC Genomics Workbench 6.5.1 RNA-Seq tool with default parameters. An artificial nonself ‘donor’ sample was also generated by combining the Donor A and B reads in the mapping stage, to provide a baseline to which to compare the results of subsequent allografts. Two count matrices were generated, with columns corresponding to different samples and rows to the Aqu2.1 gene models; the first table showed RPKM (reads per kilobase of transcript per million mapped reads) values for PCA, which was performed using BLIND [47], and the second showed total gene-wise read counts for differential expression analysis.

### 4.5. Independent Filtering and Differential Gene Expression Analysis

Independent filtering was performed to remove statistically uninformative genes and improve multiple testing correction outcomes [48], using the Bioconductor packages genefilter v1.46.1 [49] and DESeq v1.16.0 [50] as described in the genefilter vignette [49]. The bottom 50% of genes, as ranked by total genewise counts across samples, were removed accordingly. Differential gene expression (DGE) analysis was performed using EdgeR version 3.6.8 [51,52,53,54]. To help compensate for the lack of available replication, a reduced experimental model was generated in which the within-time course samples were grouped together, and the common dispersion across all genes was calculated using this model (common dispersion = 0.1606744). The analysis was then re-run with the full explanatory model, where samples were grouped by treatment (AA, BB or AB) and time (0 hpg to 72 hpg). The common dispersion value determined above was also used for this analysis. Genes exhibiting statistically significant (*p* ≤ 0.01) changes of four-fold or greater (log2) expression were identified using EdgeR’s GLM functionality.

### 4.6. Gene Ontology

GO annotation of the Aqu2.1 gene models was performed using Blast2GO version 2.8 [55] and annotations were manually reformatted for downstream analysis. BiNGO [56] was run with default parameters to identify Biological Process and Molecular Function GO terms that were statistically significantly over-enriched in the gene lists of interest, relative to the rest of the *A. queenslandica* genome. Enriched GO terms were clustered based on semantic similarity (SimRel measure) using REVIGO [57]. Similar GO terms with a redundancy of >0.7 were collapsed. Gene counts per enriched GO term were used to determine treemap layouts.

### 4.7. Graphics

Venn diagrams were generated using the online tool Venny [58]. Unscaled heat maps showing log_2_ fold changes between genes of interest were generated using the R function heatmap.2 within the gplots package [59] using default clustering parameters.

## 5. Conclusions

The process of self-nonself grafting in *A. queenslandica* appears to occur over three days. Self grafts initiated fusion between 12 to 48 hpg, and the graft interface had nearly completely disappeared by 72 hpg. The nonself graft response does not appear to be aggressive (e.g., involving the attack of one graft partner). Instead, both partners remain alive and healthy for the duration of the graft response. Allografts may undergo a period of transitory fusion between 12 and 48 hpg, where weak bonds formed between the sponge pieces, possibly to allow direct cell-cell contact between the rejecting sponges. Analysis of the global transcriptional changes occurring during this time suggests that there is not a large degree of defensive gene activation in the vicinity of the allograft, instead normal cell physiological processes, communication and proliferation appear to be repressed.

## Figures and Tables

**Table 1:** Self and nonself graft response scoring.

Time Point	Self	Nonself
12 hpg ^1^	4/2/2 ^2^	0/0/4
24 hpg	6/1/1	2/2/0
48 hpg	8/0/0	0/0/4
72 hpg	8/0/0	0/0/4

^1^ hours post grafting; ^2^ fusion/ambiguous/rejection.

**Table 2:** Transcriptome sequencing statistics.

Library	Total Bases	Read Count	Trimmed Read Count	GC (%)	Q20 (%)	Q30 (%)
Donor A	2,809,914,132	27,820,932	26,228,938	42.1	96.3	91.2
Donor B	2,663,155,678	26,367,878	25,007,728	41.9	96.6	91.7
T12 AA	2,435,185,144	24,110,744	22,538,612	41.4	95.9	90.6
T12 BB	2,494,153,186	24,694,586	23,222,990	41.9	96.2	91.1
T12 AB	2,682,231,144	26,556,744	24,878,304	40.1	96.3	91.2
T24 AA	2,229,386,534	22,073,134	20,895,012	41.5	96.5	91.5
T24 BB	2,249,828,934	22,275,534	20,912,980	41.1	96.2	91.0
T24 AB (A)	2,109,872,022	20,889,822	19,686,010	42.1	96.3	91.0
T24 AB (B)	2,006,084,624	19,862,224	18,581,488	42.0	95.9	90.5
T24 AB (C)	1,762,637,456	17,451,856	16,136,524	41.7	95.4	89.5
T48 AA	2,475,671,196	24,511,596	23,032,818	41.5	96.2	91.1
T48 BB	2,356,682,894	23,333,494	21,971,450	40.9	96.4	91.3
T48 AB	2,041,949,926	20,217,326	19,135,330	43.1	96.4	91.2
T72 AA	2,277,699,076	22,551,476	21,139,356	41.1	96.1	90.9
T72 BB	2,421,596,402	23,976,202	22,378,750	42.1	95.8	90.3
T72 AB	2,477,912,992	24,533,792	23,158,336	42.0	96.4	91.4

## Supplementary Materials

The following are available online at www.mdpi.com/1660-3397/15/5/136/s1, Figure S1: Statistically significant enriched gene ontology terms in the nonself time course, Table S1: List of Aqu2.1 accession numbers of four-fold or higher differentially expressed genes.
