# Supplementary material for: Transcriptomic Profiling of the Allorecognition Response to Grafting in the Demosponge Amphimedon queenslandica
Source: Mar Drugs. 2017 May 11;15(5):136. doi: 10.3390/md15050136 (PMC5450542; doi:10.3390/md15050136)
Supplement: Supplementary file 1 [file marinedrugs-15-00136-s001.zip › supplementary/FigureS1.pdf]

Figure S1. Enriched Gene Ontology terms in the nonself timecourse. Each treemap represents the statistically enriched Gene Ontology (GO) terms (for Biological Process and Molecular Function terms) associated with the genes which are up- or downregulated at different times in the nonself graft time course. The total number of up- or downregulated genes for each time point is given at the top of each page. Within each treemap, each coloured box represents an enriched GO term associated with the gene list, with box size proportional to the number of genes annotated with that GO term (also shown in brackets). Identically-coloured boxes represent superclusters of loosely related GO.

0 hpg vs 12 hpg - Downregulation (n = 379)

Molecular Function

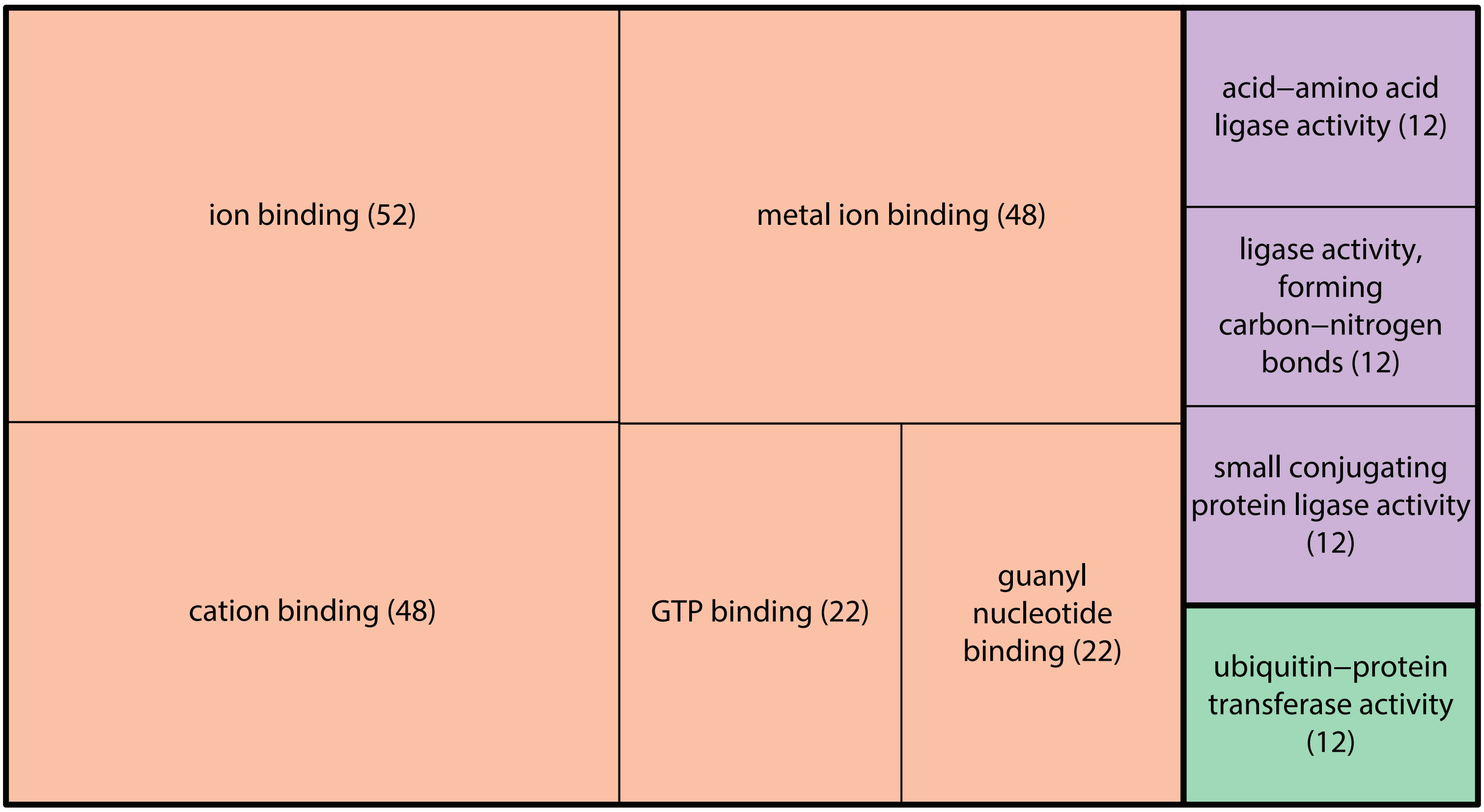

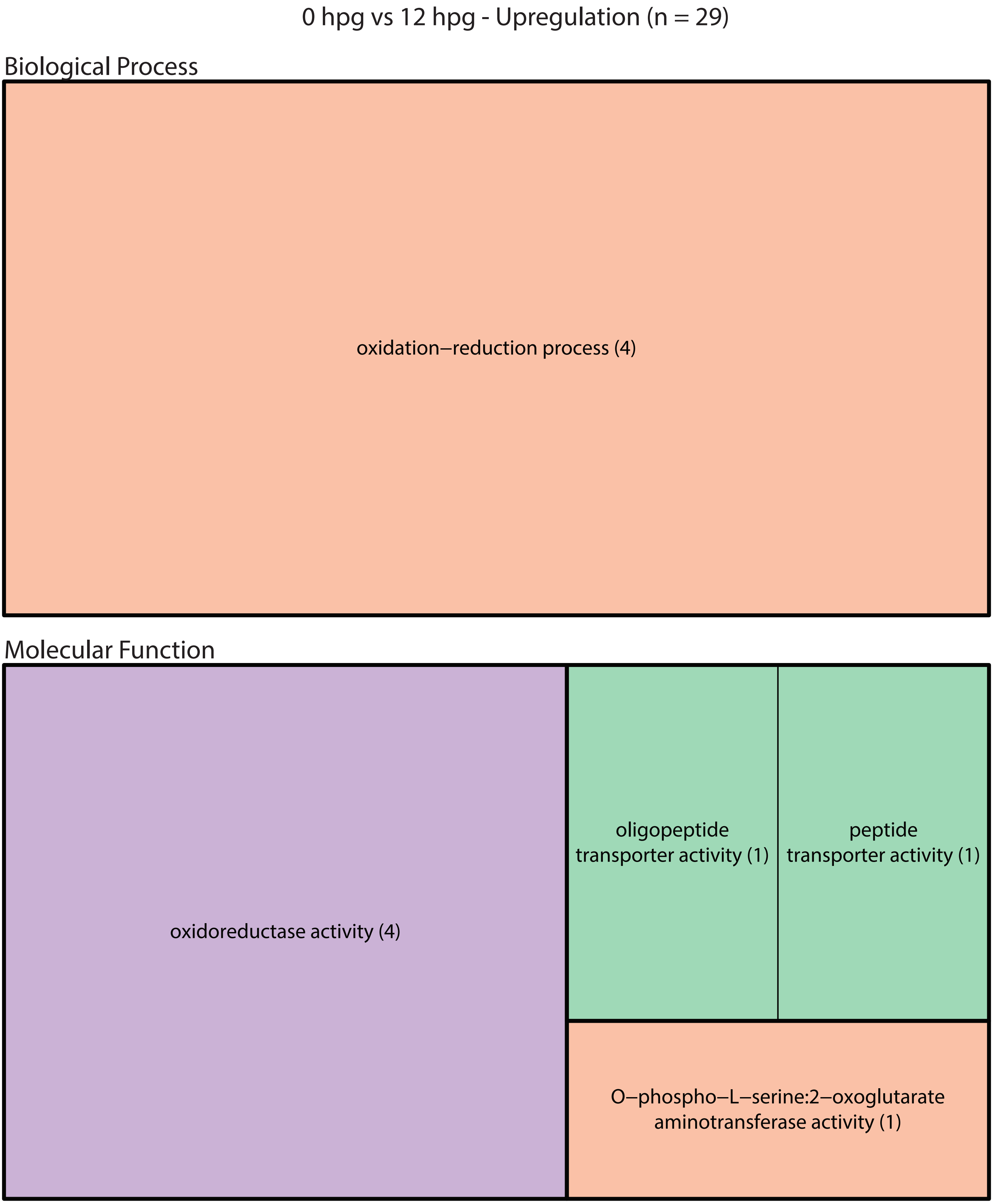
0 hpg vs 12 hpg - Upregulation (n = 29)
Biological Process
oxidation−reduction process (4)
Molecular Function
oxidoreductase activity (4)
oligopeptide transporter activity (1)
peptide transporter activity (1)
O−phospho−L−serine:2−oxoglutarate aminotransferase activity (1)

12 hpg vs 24 hpg - Downregulation (n = 101) - 1 of 2

Biological Process

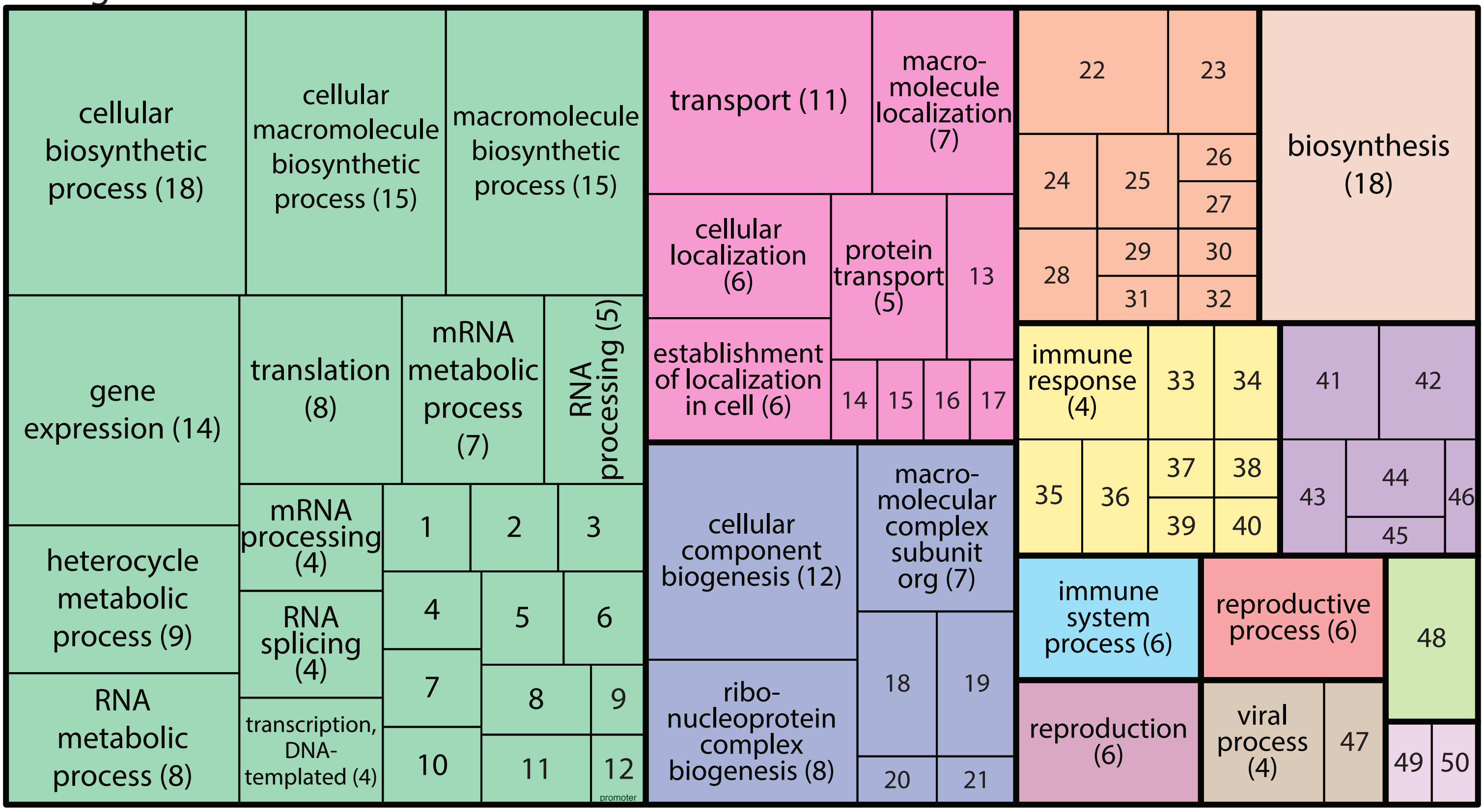

1 - 7–methylguanosine RNA capping (2)
2 - DNA-templated transcription, elongation (2)
3 - DNA-templated transcription, termination (2)
4 - RNA secondary structure unwinding (2)
5 - transcription elongation from RNA pol. III promoter (2)
6 - transcription from RNA pol. III promoter (2)
7 - termination of RNA pol. III transcription (2)
8 - transcription initiation from RNA pol. II promoter (2)
10 - transcription elongation from RNA pol. II promoter (2)
9 - proteasomal ubiquitin-independent protein catabolic process (1)
11 - translational initiation (2)
12 - regulation of transcription from RNA pol. promoter (1)
13 - nucleobase–containing compound transport (3)
14 - acetyl–CoA transport (1)
15 - coenzyme transport (1)
16 - rRNA export from nucleus (1)
17 - rRNA transport (1)
18 - cellular component disassembly (3)
19 - protein complex disassembly (3)
20 - desmosome assembly (1)
21 - histome H3-T6 phosphorylation (1)
22 - regulation of multicellular organismal process (5)
23 - positive regulation of multicellular organismal process (3)
24 - germ cell development (2)
25 - regulation of type I interferon production (2)
26 - activation of protein kinase A activity (1)
27 - negative regulation of glucose import (1)
28 - regulation of cell cycle arrest (2)
29 - positive regulation of behaviour (1)
30 - positive regulation of inflammatory response (1)
31 - positive regulation of cardiac muscle hypertrophy (1)
32 - regulation of macrophage differentiation (1)
33 - response to peptide hormone (2)
34 - transcription-coupled nucleotide excision repair (2)
35 - antigen processing and presentation of exogenous antigen (2)
37 - cellular response to parathyroid hormone stimulus (1)
36 - antigen processing & presentation of exogenous peptide antigen (2)
38 - response to mercury ion (1)
39 - neutrophil chemotaxis (1)
40 - response to parathyroid hormone (1)
41 - nucleobase metabolic process (3)
42 - purine nucleobase metabolic process (3)
43 - ether metabolic process (2)
44 - glycerol ether metabolic process (2)
45 - creatine biosynthetic process (1)
46 - creatine metabolic process (1)
47 - regulation of viral process (2)
48 - multi-organism process (4)
49 - triglyceride catabolism (1)
50 - glycrolipid catabolism (1)

12 hpg vs 24 hpg - Downregulation (n = 101) - 2 of 2

Molecular Function

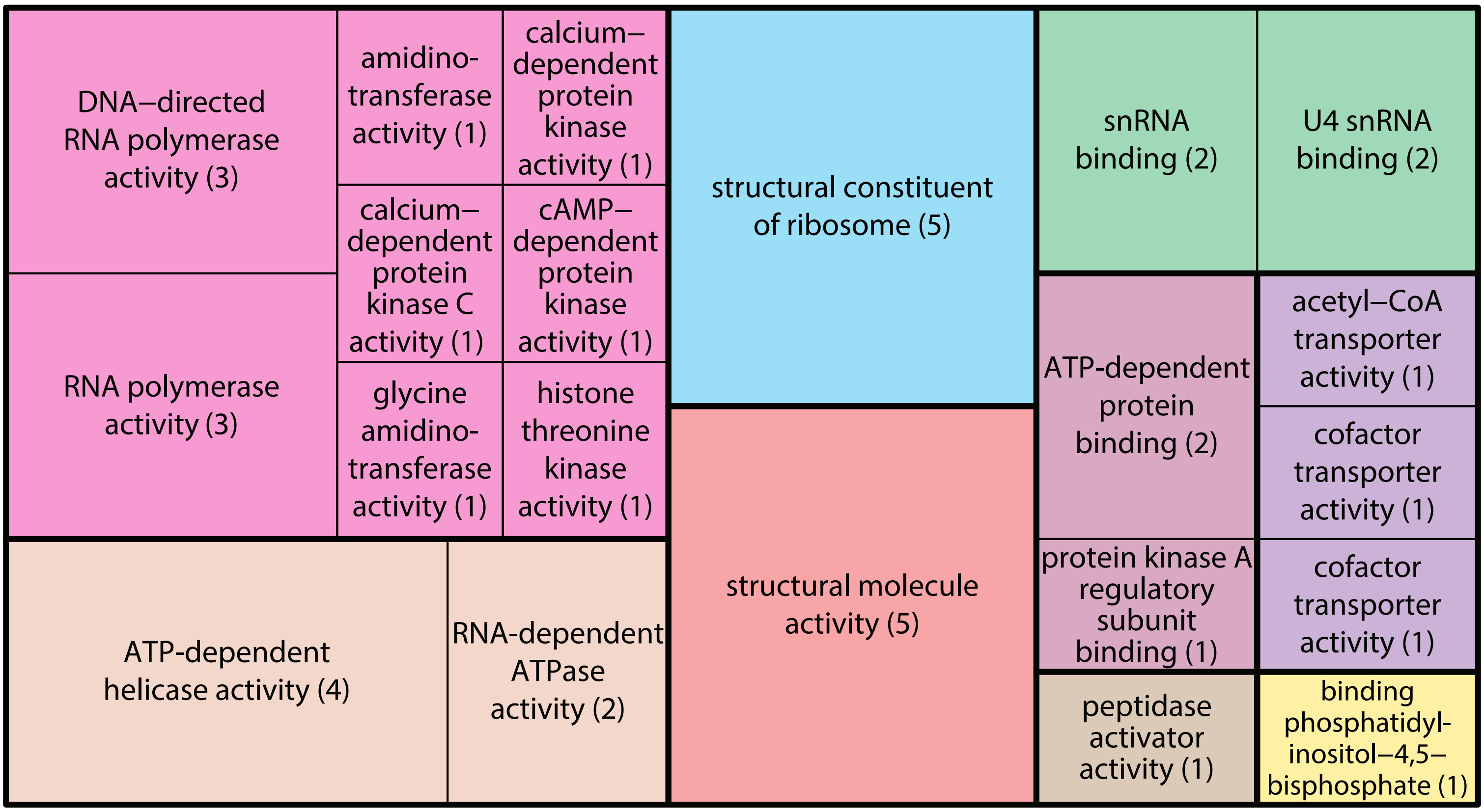

12 hpg vs 24 hpg - Upregulation (n = 9)

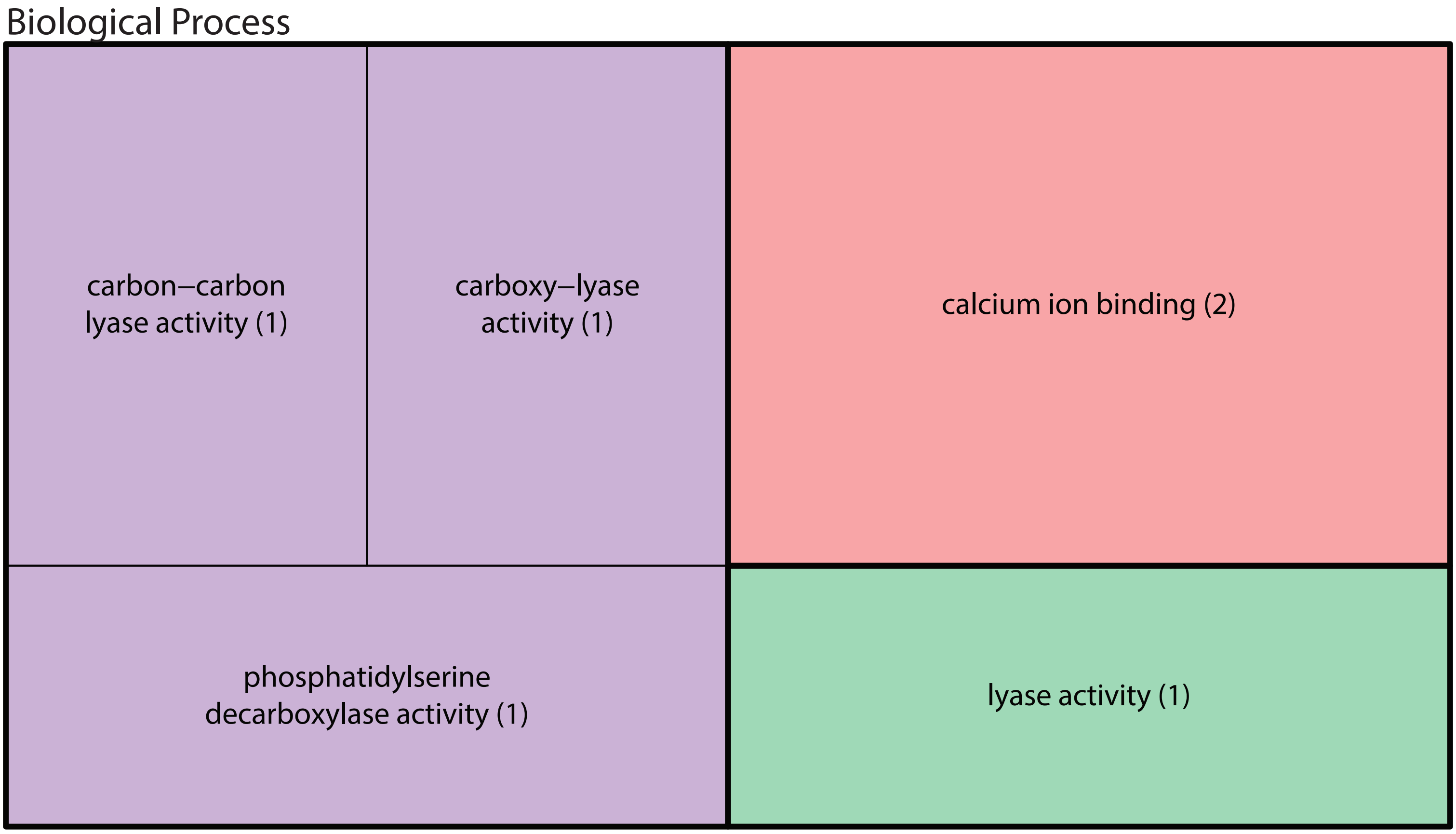

24 hpg vs 48 hpg - Downregulation (n = 1178)

Biological Process

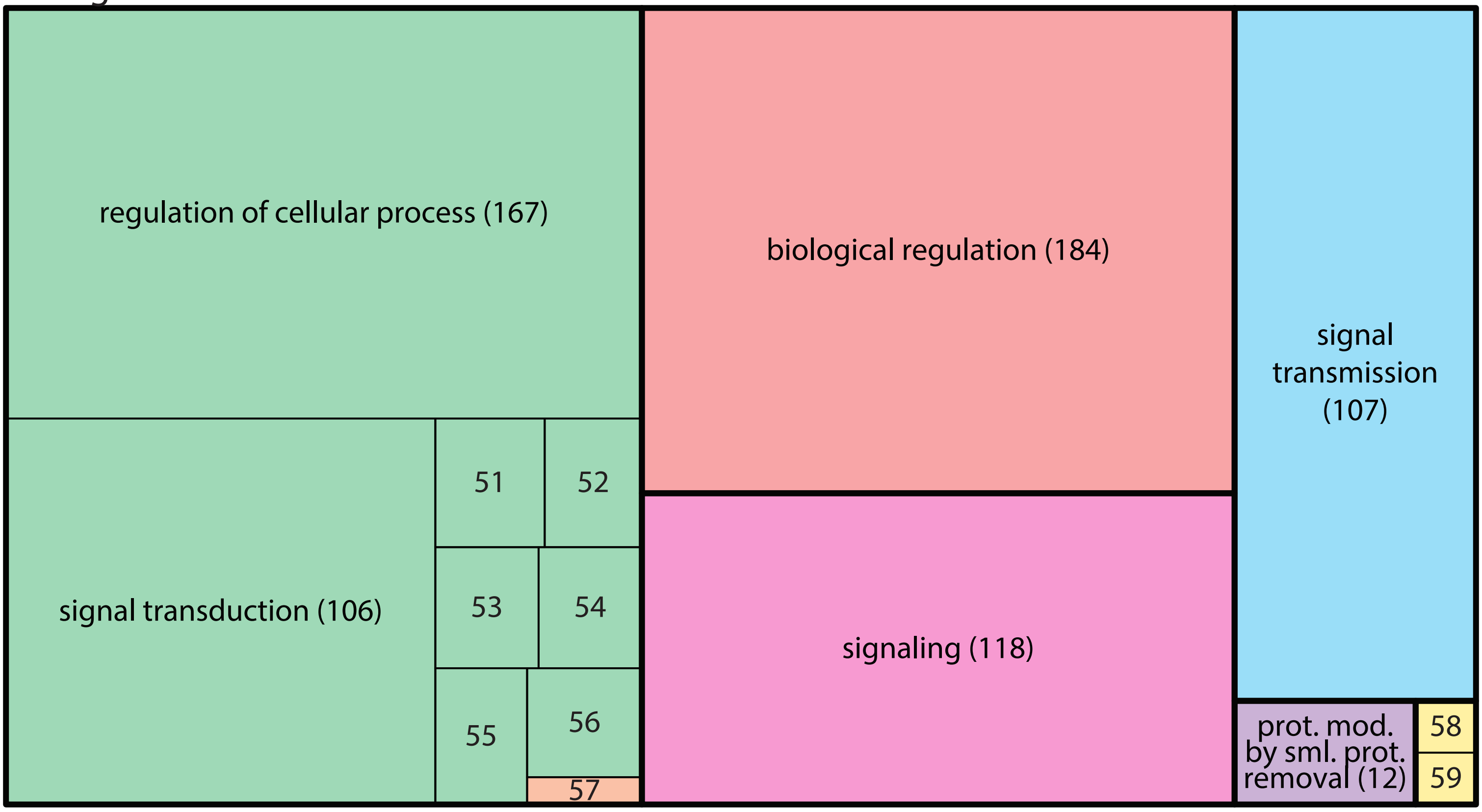

Molecular Function

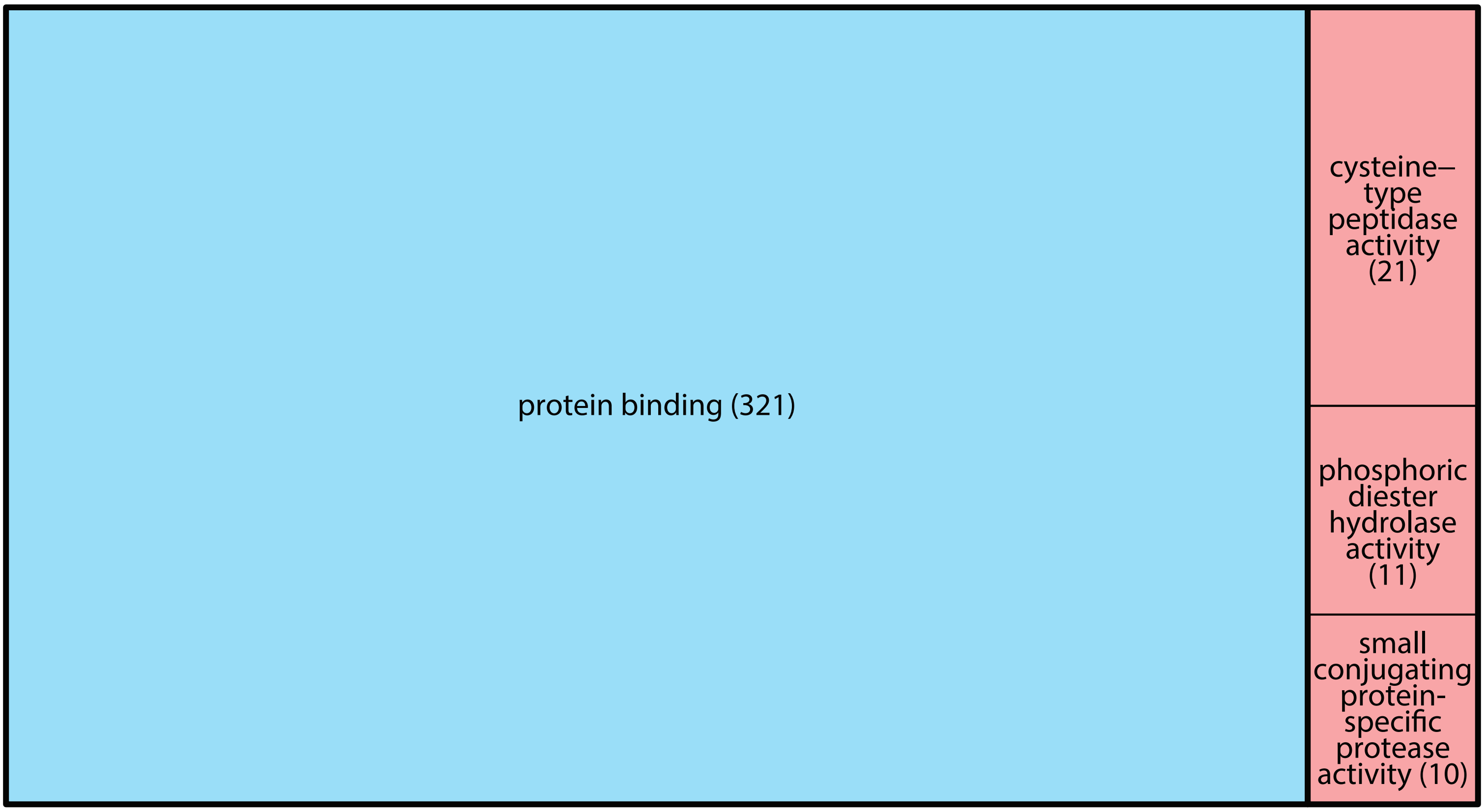

51 - regulation of cysteine-type endopeptidase activity involved in apoptotic process (9)
52 - base-excision repair (8)
53 - negative regulation of cell cycle process (8)
54 - negative regulation of mitotic cell cycle (8)
55 - positive regulation of cell growth (8)
56 - positive regulation of cell size (8)
57 - synaptonemal complex organisation (2)
58 - oocyte maturation (2)
59 - synaptonemal complex assembly (2)

24 hpg vs 48 hpg - Upregulation (n = 1049)

Biological Process

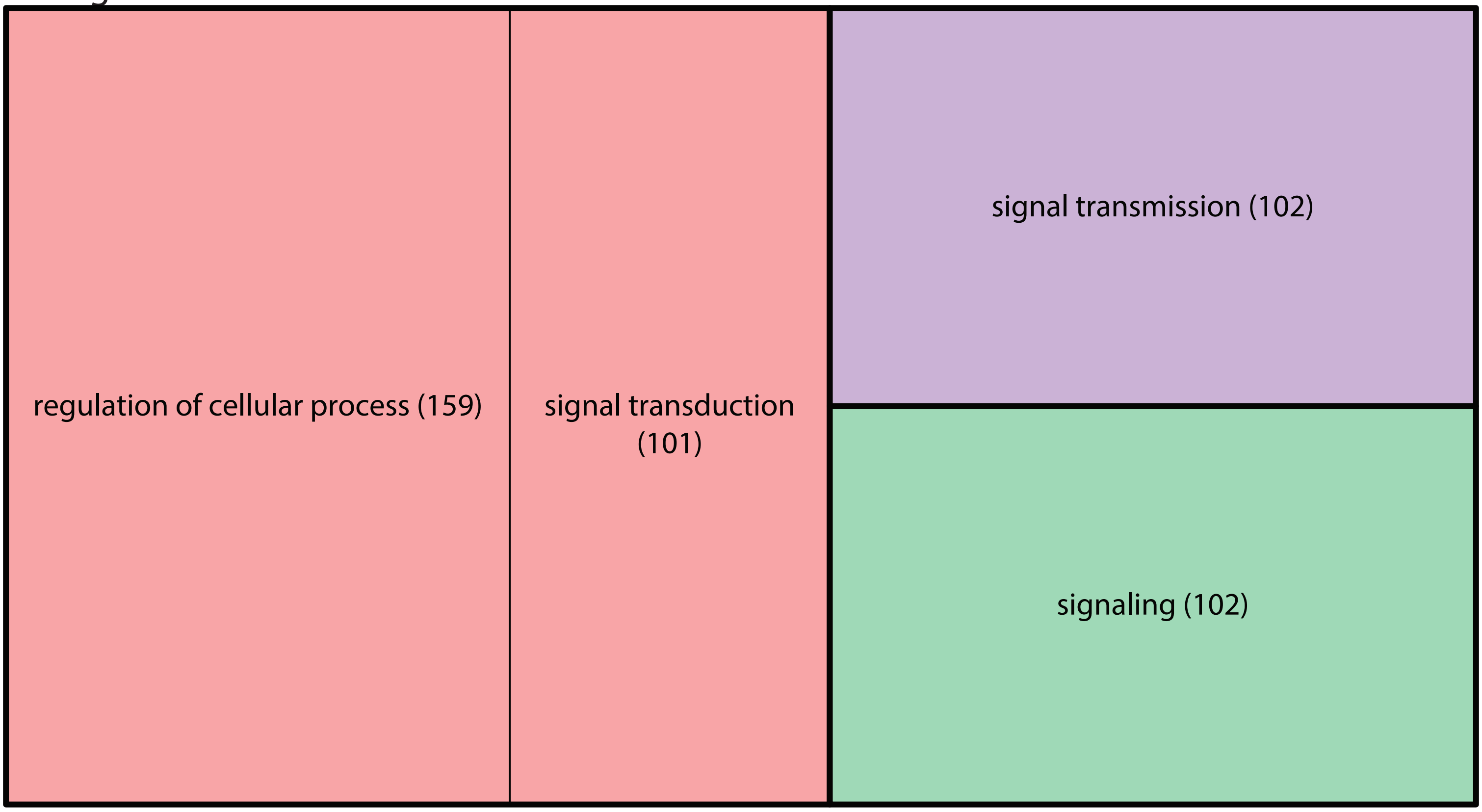

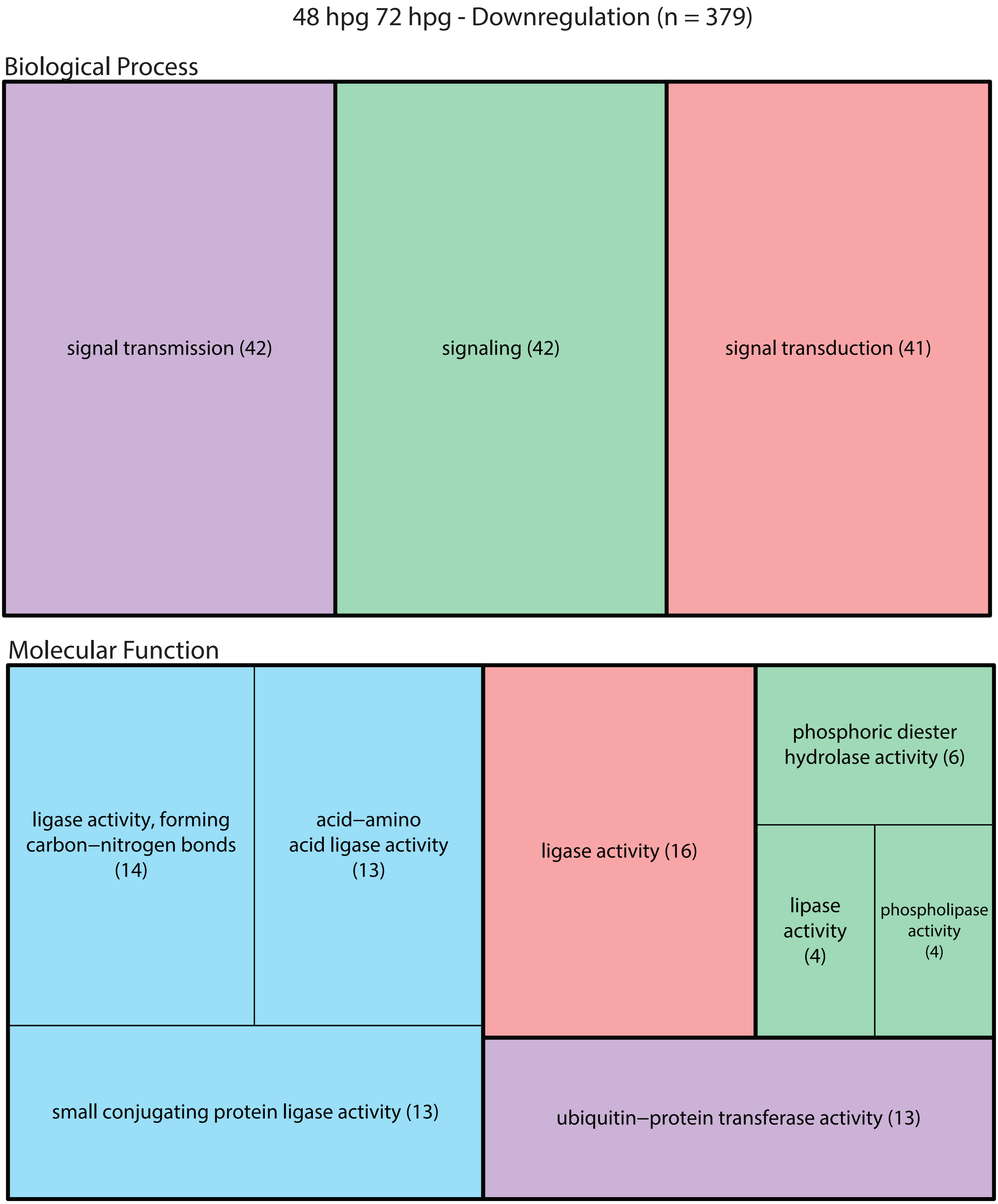
48 hpg 72 hpg - Downregulation (n = 379)
Biological Process
signal transmission (42)
signaling (42)
signal transduction (41)
Molecular Function
ligase activity, forming carbon–nitrogen bonds (14)
acid–amino acid ligase activity (13)
ligase activity (16)
phosphoric diester hydrolase activity (6)
lipase activity (4)
phospholipase activity (4)
small conjugating protein ligase activity (13)
ubiquitin–protein transferase activity (13)

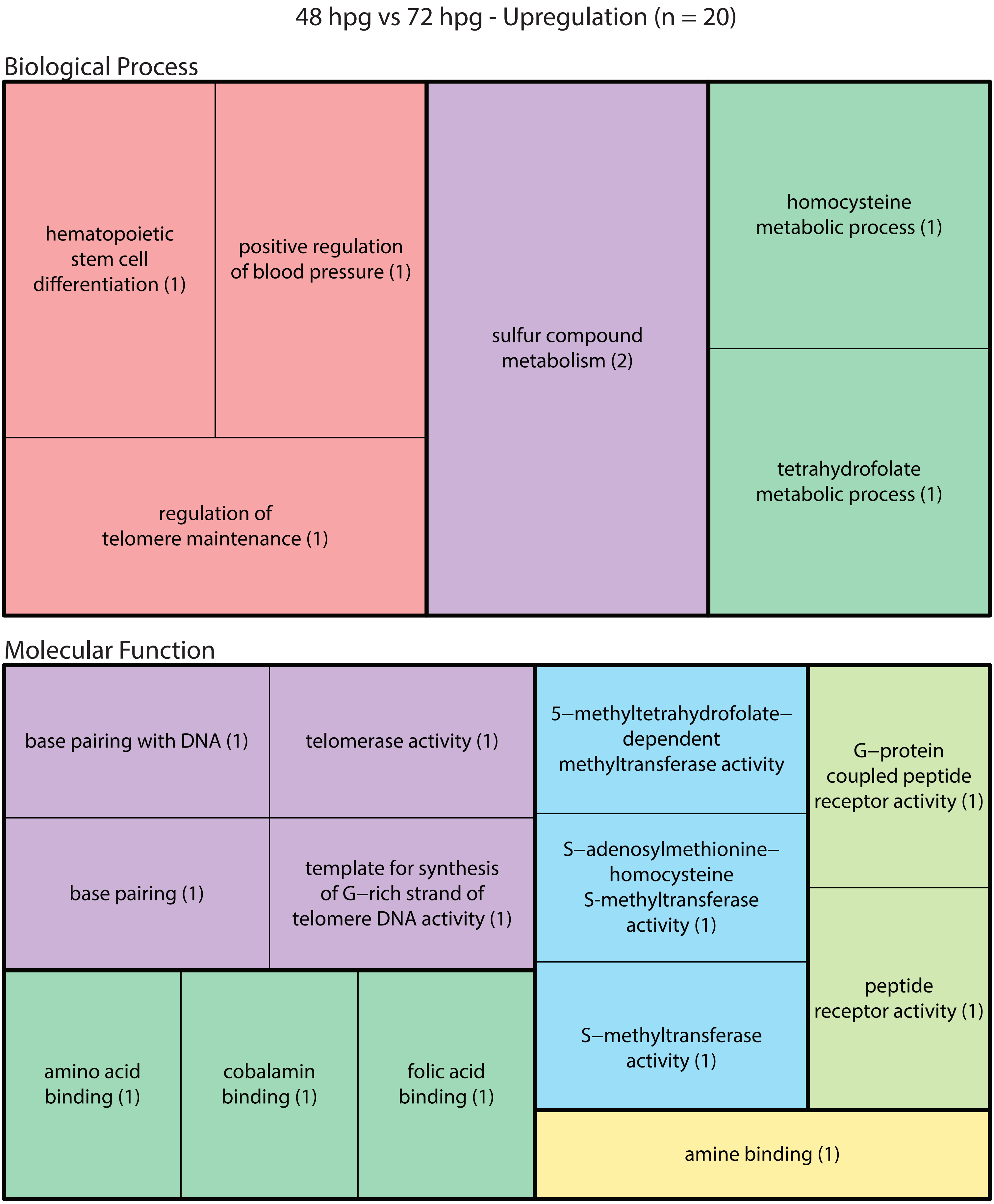
48 hpg vs 72 hpg - Upregulation (n = 20)
Biological Process
hematopoietic stem cell differentiation (1)
positive regulation of blood pressure (1)
regulation of telomere maintenance (1)
sulfur compound metabolism (2)
homocysteine metabolic process (1)
tetrahydrofolate metabolic process (1)
Molecular Function
base pairing with DNA (1)
telomerase activity (1)
base pairing (1)
template for synthesis of G–rich strand of telomere DNA activity (1)
amino acid binding (1)
cobalamin binding (1)
folic acid binding (1)
5–methyltetrahydrofolate–dependent methyltransferase activity
S–adenosylmethionine–homocysteine S-methyltransferase activity (1)
S–methyltransferase activity (1)
G–protein coupled peptide receptor activity (1)
peptide receptor activity (1)
amine binding (1)
